# Photoinitiator Free Resins Composed of Plant-Derived Monomers for the Optical µ-3D Printing of Thermosets

**DOI:** 10.3390/polym11010116

**Published:** 2019-01-11

**Authors:** Migle Lebedevaite, Jolita Ostrauskaite, Edvinas Skliutas, Mangirdas Malinauskas

**Affiliations:** 1 Department of Polymer Chemistry and Technology, Kaunas University of Technology, Radvilenu Rd. 19, 50254 Kaunas, Lithuania; migle.lebedeveaite@ktu.lt; 2 Laser Research Center, Vilnius University, Sauletekis Ave. 10, 10223 Vilnius, Lithuania; edvinas.skliutas@ff.vu.lt (E.S.); mangirdas.malinauskas@ff.vu.lt (M.M.)

**Keywords:** acrylated epoxidized soybean oil, vanillin dimethacrylate, vanillin diacrylate, photocross-linking, direct laser writing, nanolithography, optical 3D printing, two-photon polymerization (2PP), multi-photon processing

## Abstract

In this study, acrylated epoxidized soybean oil (AESO) and mixtures of AESO and vanillin dimethacrylate (VDM) or vanillin diacrylate (VDA) were investigated as photosensitive resins for optical 3D printing without any photoinitiator and solvent. The study of photocross-linking kinetics by real-time photorheometry revealed the higher rate of photocross-linking of pure AESO than that of AESO with VDM or VDA. Through the higher yield of the insoluble fraction, better thermal and mechanical properties were obtained for the pure AESO polymer. Here, for the first time, we validate that pure AESO and mixtures of AESO and VDM can be used for 3D microstructuring by employing direct laser writing lithography technique. The smallest achieved spatial features are 1 µm with a throughput in 6900 voxels per second is obtained. The plant-derived resins were laser polymerized using ultrashort pulses by multiphoton absorption and avalanche induced cross-linking without the usage of any photoinitiator. This advances the light-based additive manufacturing towards the 3D processing of pure cross-linkable renewable materials.

## 1. Introduction

In recent years, 3D printing or rapid prototyping as a flexible additive manufacturing technique became very popular because of its simplicity, relatively low cost, and unlimited creativity. This process enables the creation of complex three-dimensional objects which cannot be cut, assembled or carved. It is possible because of Computer-Aided Design (CAD) modeling, where various objects are generated and files are transmitted for the printing of 3D items [1]. Stereolithography (SLA) is a process which obtains a high printing accuracy and speed, simple and low raw material usage technology [2]. It is a layer by layer photopolymerization method, where the photosensitive resin is polymerized by UV/VIS light.

Epoxy and acrylic resins are the most popular materials in optical 3D printing. Printed epoxy polymers have low shrinkage and high structural stability, while acrylates have high light sensitivity, low critical energy and viscosity, controllable mechanical properties and relatively high dependence on temperature and humidity changes [3]. While epoxides have higher structural stability, acrylates possess higher photosensitivity due to which they are more popular in the SLA process.

Most of the photosensitive resins for optical 3D printing are made from acrylic oligomers, acrylic monomers and/or reactive diluents, photoinitiator and UV stabilizators/blockers [4]. Photopolymerized acrylates are in an irregular molecular structure because of high curing speed. Petroleum-derived acrylic resins such as polyesters, polyether oligomers or diglicidylether bisphenol A acrylates are those that are mostly used for optical 3D printing [5]. Due to decreasing petroleum recourses, it became crucial to search for alternative materials such as renewable resources [6]. Natural oils are one of the best alternatives for petroleum-derived resins [7,8,9]. Due to their richness in double bonds which can be polymerized or converted to other functional groups, biodegradability and renewability, natural oils became a popular target of researchers [10,11].

Soybean oil is one of the most promising materials to replace petroleum-derived resins. It contains a high amount of unsaturated fatty acids such as monounsaturated oleic acid (C-18:1, ~23%), polyunsaturated α-linoleic acid (C-18:3, 7–10%), and linoleic acid (C-18:2, ~51%) [12,13]. Carbon-carbon double bonds of fatty acids can be oxidized [14], polymerized [15,16] or converted to various reactive groups [17,18,19].

Acrylated epoxidized soybean oil (AESO) is produced by the epoxidation of fatty acid double bonds followed by epoxy ring acrylation [20,21,22,23]. Due to the high amount of various functional groups such as the acrylic, epoxy and hydroxy groups, AESO is widely used in industry and is commercially available under Ebecryl 860 trademark [24]. AESO can be polymerized by UV/VIS light using appropriate photoinitiators and can form a cross-linked polymer network. AESO is already photopolymerized with polycaprolactone diacrylate and polyethylenglycol diacrylate [25], tetrahydrophurphuryl acrylate [26,27], myrcene [28], and various thiols [29,30]. The addition of comonomers improved the mechanical and thermal properties as well as solvent resistance of the resulted polymers. Due to AESO fatty acid long aliphatic chains, cross-linked polymers are soft and mechanically resistless. To improve the polymer mechanical properties, aromatic comonomers are added. Such compounds such as styrene [31,32], divinylbenzene (DVB) [33,34], dicyclopentadiene [35] and acrylepoxymethylester [36] were used as stiffening agents for AESO polymers. But these materials are petroleum-derived and harmful to health [37,38,39]. Initially, we selected the plant-derived vanillin acrylates to replace the petroleum-derived aromatic compounds because of their aromatic structure, renewability and reactivity [40].

Vanillin dimethacrylate (VDM) or methacrylated vanillin alcohol is produced from lignin, one of the most abundant natural polymers [41]. The bio-based thermosets made from VDM and maleinated AESO showed high glass transition temperatures (63–79 °C) and high Young’s modulus values (570–855 MPa), but a high viscosity of the mixture and very long reaction time (about 8 h) was also observed [42]. Vanillin diacrylate (VDA) is a bifunctional aromatic compound which can also be produced from lignin. It has two acrylic groups which can be polymerized via free-radical polymerization, yet no data was found of its usage in polymerization.

**Scheme 1:**
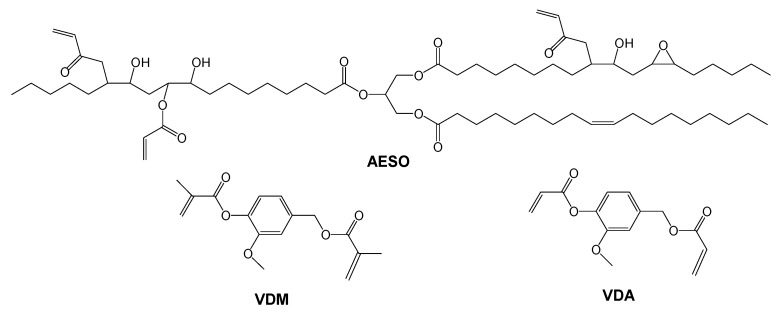
The chemical structure of acrylated epoxidized soybean oil (AESO), vanillin dimethacrylate (VDM) and vanillin diacrylate (VDA).

In this study, the plant-derived AESO, VDM and VDA (Scheme 1) were used as photosensitive monomers for the optical 3D printing of thermosets. The monitoring of photocross-linking kinetics was carried out by real-time photorheometry which provides a wide range of information on typical rheological properties such as viscosity and shear modulus while the material is irradiated with UV/VIS light. It enables us to track the occurrence of structural phenomena, such as gelation and vitrification [43], indicating the moment, when the structural changes have started [44]. The chemical structure of the polymers was investigated by FTIR spectroscopy. The formation of polymer cross-linked structure was confirmed by Soxhlet extraction. The thermal properties of cross-linked polymers were investigated by differential scanning spectroscopy and thermogravimetric analysis. The compressive modulus was determined from the top pressure test. The plant-derived resins AESO and AESO/VDM were laser polymerized using ultrashort pulses by multiphoton absorption and avalanche induced cross-linking without a photoinitiator.

## 2. Materials and Methods

### 2.1. Materials

Acrylated epoxidized soybean oil (AESO, an average number of acryloyl groups per molecule calculated from the ^1^H-NMR spectrum is 2.7 and 0.3 of epoxide groups) was purchased from Sigma-Aldrich (Darmstadt, Germany). Vanillin dimethacrylate (VDM) and vanillin diacrylate (VDA) were purchased from Specific Polymers. Chloroform was purchased from Chempur (Karlsruhe, Germany). All materials were used without further purification.

### 2.2. Real-Time Photorheometry

UV/VIS curing tests of pure AESO and two resin series, AESO/VDM and AESO/VDA (Table 1), without any photoinitiator and solvent were carried out with an MCR302 rheometer from Anton Paar (Graz, Austria) equipped with plate/plate measuring system. A Peltier-controlled temperature chamber with the glass plate (diameter of 38 mm) and the top plate PP08 (diameter of 8 mm) was used. The measuring gap was set to 0.3 mm. The samples were irradiated at room temperature by UV/VIS radiation in a wavelength range of (250–450) nm through the glass plate of the temperature chamber using a UV/VIS spot curing system OmniCure S2000, Lumen Dynamics Group Inc (Mississauga, Ontario, Canada). The intensity of the irradiation was 9.3 W·cm^−2^ (high pressure 200 W mercury vapor short arc). Shear mode with a frequency of 10 Hz and a strain of 0.3% was used. Storage modulus G’, loss modulus G”, loss factor tanδ (tanδ = G”/G’), and complex viscosity η* were recorded as a function of irradiation time. The onset of UV/VIS irradiation was at 60 s after the experiment start for all samples.

### 2.3. Preparation of Cross-Linked Polymers

The homopolymer pAESO was synthesized from pure AESO and the copolymer series pAESO/VDM was synthesized from the mixtures of AESO and VDM without a photoinitiator and any solvent. The mixtures of AESO and VDM (Table 1) were stirred at 40 °C for 24 h. Resins were poured into a tablet-shaped (⦰ 15 mm, h 3 mm) Teflon mold and irradiated with Helios Italquartz, model GR.E 500 W lamp with an intensity of 310 mW·cm^−2^ at the distance of 15 cm until hard polymer tablets were formed.

### 2.4. Chemical Structure Analysis

Fourier Transform Infrared Spectroscopy (FT-IR) measurements of photocross-linked polymer samples were performed on a Spectrum BX II FT-IR spectrometer (Perkin Elmer, Llantrisant, UK). The spectra were acquired from 10 scans. The range of the wavenumber was (400–4000) cm^−1^.

### 2.5. Soxhlet Extraction

The amount of the insoluble fraction was determined by Soxhlet extraction. Samples of photocross-linked polymers were put into a filter package and placed in a Soxhlet apparatus (Sigma-Aldrich, Darmstadt, Germany). Extraction was performed with chloroform for 24 h. Insoluble fractions were dried under vacuum to a constant weight. The amount of insoluble fraction was calculated as the difference of the sample weight before and after extraction.

### 2.6. Differential Scanning Calorimetry

The glass transition temperature (T_g_) of the photocross-linked polymers were estimated by differential scanning calorimetry (DSC). The measurements were performed on a DSC 8500 apparatus (Perkin Elmer, Llantrisant, UK) with a heating-cooling-heating rate of 10 °C·min^−1^ under a nitrogen atmosphere (nitrogen flow rate 50 mL·min^−1^). The T_g_ value was taken as the middle point in the heat capacity step of the glass transition.

### 2.7. Thermogravimetric Analysis

The thermal stability of prepared polymers was determined by thermogravimetric analysis (TGA). The measurements were performed on a TGA 4000 apparatus (Perkin Elmer, Llantrisant, UK) in a temperature range from room temperature to 800 °C at a heating rate of 20 °C·min^−1^ under a nitrogen atmosphere (nitrogen flow rate 100 mL·min^−1^).

### 2.8. Mechanical Testing

The mechanical properties of the photocross-linked polymer tablets were estimated by a compression test on a BDO-FB0.5TH (Zwick/Roell, Kennesaw, Georgia, USA) testing machine at room temperature. The cross-linked polymer specimen with a 15 mm diameter and 3 mm thickness was placed in a Teflon mold of the same size in order to avoid the expansion of specimen to the sides during the test. The specimen was pressed with a cylindrical steel rod with a flat end diameter of 8 mm. The speed of the rod movement was 5 mm/min. The specimen pressure was stopped when the upper force limit of 100 N was reached. The compressive modulus was calculated by the following equation:

(1) $$E_{c}=\frac{F\cdot l_{0}}{S\cdot ∆l}$$

where *E_c_* is a compressive modulus (N/mm^2^); *F* is a force (N); *S* is a surface area of the specimen that interacts with the steel rod flat end (mm^2^); *l*_0_ is an initial thickness of the specimen (mm); Δ*l* is the difference of the initial thickness of the specimen and the thickness of a loaded specimen (mm).

The mean values of the ten samples of each polymer were calculated. Results with a within-group variation below 5% were taken.

### 2.9. Direct Laser Writing 3D Lithography

**Figure 1:**
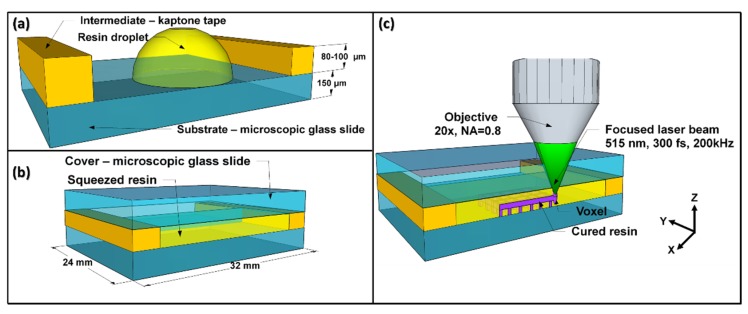
The explanatory scheme of a sample preparation and Direct Laser Writing (DLW) 3D lithography: (**a**) Kapton tape attached to the glass slide and working as an intermediate, resin’s droplet drop cast on such a substrate; (**b**) another glass slide used as a cover to squeeze the droplet and spread it uniformly through the substrate; (**c**) laser beam focused through the cover glass into the resin and initiating the 3D confined polymerization reaction.

Direct laser writing (DLW) 3D lithography experiments were conducted employing a Pharos laser (515 nm, 300 fs, 200 kHz, Light Conversion Ltd, Vilnius, Lithuania), 20 × NA = 0.8 objective and the combined movement of the linear stages and Galvano-scanners. A detailed description of the experimental setup can be found in a previous publication [45]. The goal was to figure out if AESO and VDM monomer based resins can be suitable for ultra-fast laser pulses initiated by 3D polymerization in a confined space. An investigation test to assess the optimal fabrication parameters was performed. A 3D model of so-called resolution bridges (RB) was programmed accordingly. It consisted of five rectangle-shaped columns which were 15 µm wide, 60 µm long and 15 µm high. Gaps between the columns varied from 5 µm to 20 µm every 5 µm. The five straight lines perpendicular to the columnar long edges formed in the gaps. Such a structure corresponds to the RB suspended between the columns. Each line was polymerized from a single laser beam scan. RB were obtained with different longitudinal and lateral sizes by varying the laser power (*P*), which corresponded to the light intensity (*I*) at the sample and the scanning velocity (*v*). It enabled the evaluation of the smallest features and their dependence on the applied *I* which could be formed in AESO and VDM based resins. The capability to form 3D microporous woodpile structures was demonstrated. They consisted out of two layers separated via vertical 20 µm height columns. The layers were made of a 15 µm wide and 75 µm long log pattern with a 15 µm gap between the logs, resulting in a 30 µm period. Each log was made from multiple scans whose number depended from the distance *d_xy_* between scans. *d_xy_* was 0.25–2 µm every 0.25 µm. During the fabrication, the resin was placed between two glass slides, as shown in Figure 1. After the exposure the samples were developed in 4-methyl-2-pentanone for 15 min, removing the uncured resin and leaving only the formed structures on the substrate. The fabricated structures were characterized using a scanning electron microscope (SEM, Hitachi TM-1000, Tokyo, Japan).

## 3. Results

### 3.1. Kinetics of Photocross-Linking

In this study, the photocross-linking of AESO and the mixtures of AESO and VDM or AESO and VDA was performed without any photoinitiator and solvent. The composition of resins is presented in Table 1. Different ratios of AESO and VDM or VDA were used in the resin series AESO/VDM and AESO/VDA. The resin AESO/VDM1 contains the maximum amount of VDM possible to dissolve in AESO without any solvent at 40 °C. The same amounts of VDA and VDM were taken in the resin series AESO/VDA and AESO/VDM for comparison.

**Figure 2:**
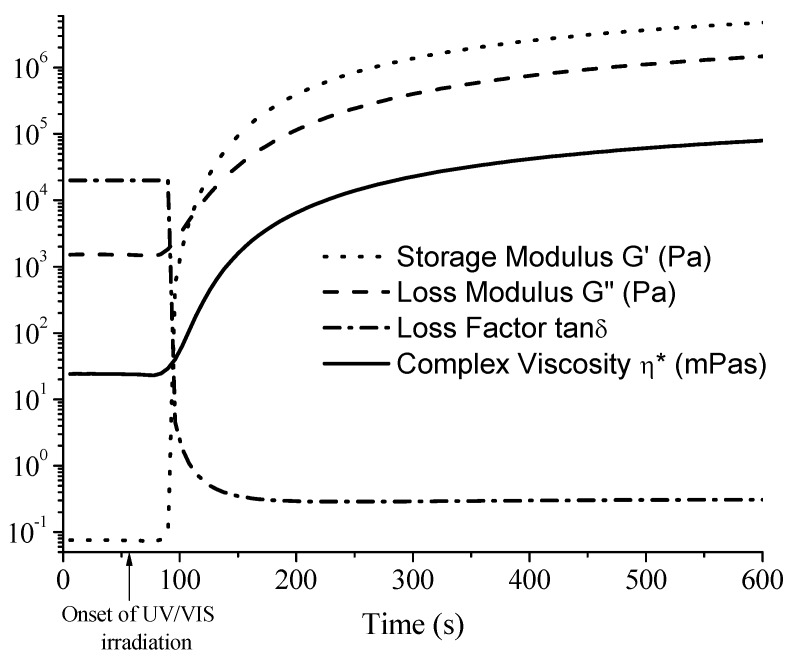
The dependencies of storage modulus G’, loss modulus G”, loss factor tanδ and complex viscosity η* of AESO on irradiation time.

The kinetics of photocross-linking was monitored by real-time photorheometry. In the photorheometry test, when the photosensitive resin is being irradiated with UV/VIS light, the values of storage modulus G’, loss modulus G”, and complex viscosity η* start to increase. This increment indicates the formation of a three-dimensional polymer network. During the process, the values of G’ increase faster and exceed the values of G” showing the high viscosity Newton liquid transforms into a hard elastic polymer. In this transformation, the point where G’ = G” is commonly used to define the gel point (t_gel_) [46]. At this point, the loss factor tanδ, the ratio of the viscous and the elastic portion of the viscoelastic deformation behavior (G”/G’) starts to decrease. Then, the values of G’ and G” increase continuously and the tanδ value decreases until the final degree of cross-linking is reached [47,48]. As an example, the dependencies of G’, G”, tanδ and η* on the irradiation time of the photocross-linking of AESO are presented in Figure 2.

**Figure 3:**
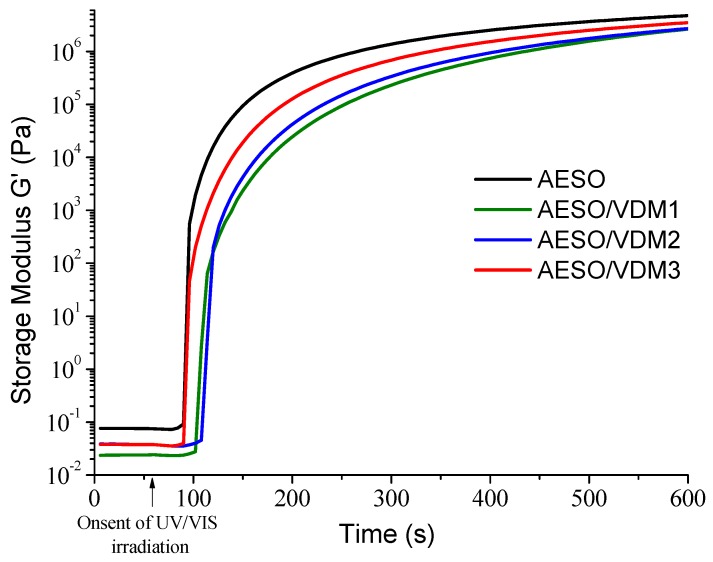
The irradiation time dependencies of the storage modulus G’ of AESO and the resin series AESO/VDM.

**Figure 4:**
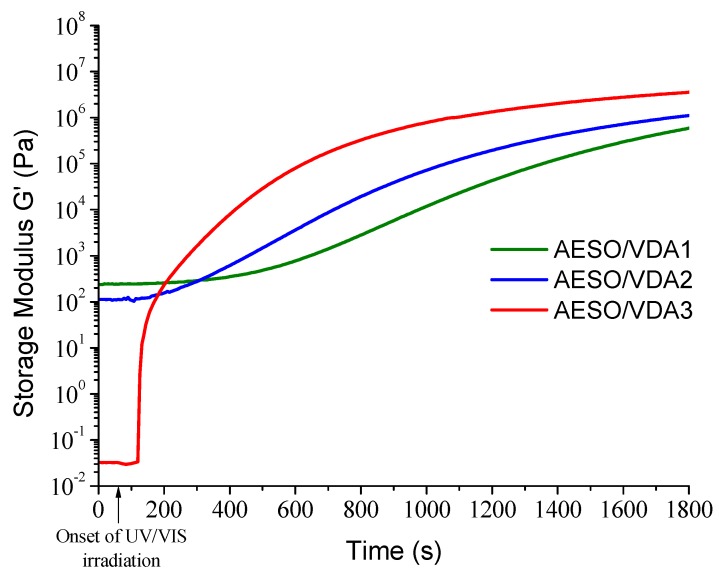
The irradiation time dependencies of the storage modulus G’ of the resin series AESO/VDA.

The values of G’, G”, η*, and t_gel_ of AESO, the resin series AESO/VDM and AESO/VDA are presented in Table 2. It was determined that the rate of photocross-linking of resins containing AESO and VDM or VDA was lower than that of pure AESO. The addition of VDM or VDA to the resins caused a longer induction period and the higher t_gel_ values. The values of t_gel_ of the resin series AESO/VDA were much higher than those of AESO or the resin series AESO/VDM. Moreover, the G’ values of AESO were higher than those of the resin series AESO/VDM and AESO/VDA, indicating the lower rigidity of the latter polymers (Figure 3 and Figure 4).

It is known that the acrylic group is more reactive than methacrylic [49]. This explains the increase of the induction period and t_gel_ value during the photocross-linking of the resin series AESO/VDM in comparison to AESO. Additionally, the slope of the G’ curve of AESO was steeper than that of the resin series AESO/VDM indicating the quicker formation of the polymer network [50]. High G’ values indicate better mechanical properties of polymers caused by the high density of cross-links [41]. Thus, the higher G’ values of AESO indicate the higher density of cross-links in this polymer (pAESO).

The irradiation time dependencies of the storage modulus of the resin series AESO/VDA are shown in Figure 4. The photocross-linking kinetics of this resin series strongly depends on the VDA concentration. The higher the amount of VDA was used, the higher the values of t_gel_ obtained (Table 1). The increase of the VDA amount caused the lower G’ values obtained to show a lower density of cross-links in these polymers. The induction period of AESO/VDA was longer and the G’ values were lower compared to those of the resin series AESO/VDM. Due to these data, especially due to the very high t_gel_ values of the resin series AESO/VDA, further experiments were performed only with AESO and the resin series AESO/VDM.

### 3.2. Characterization of Photocross-Linked Polymer Structure

**Figure 5:**
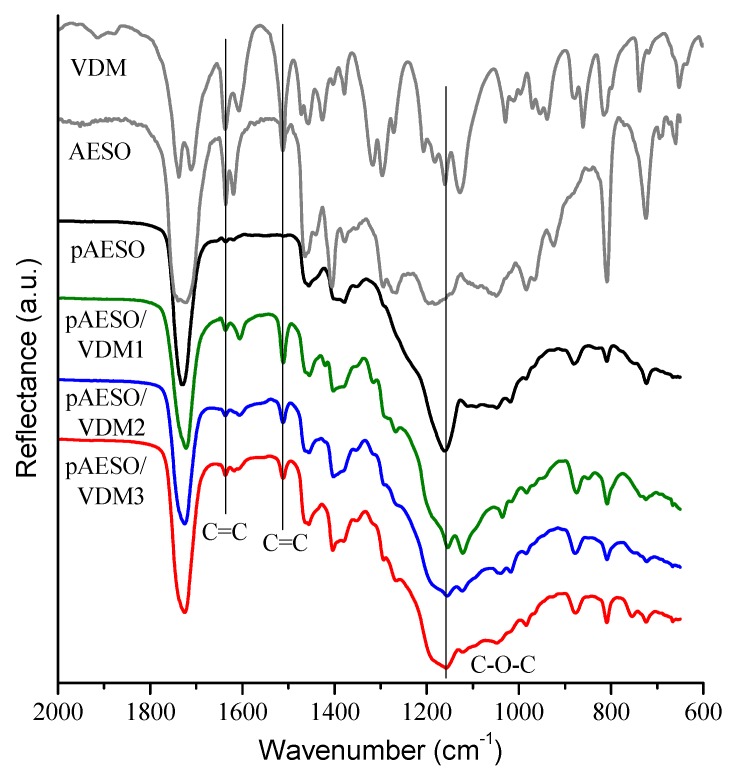
The FT-IR spectra of AESO, VDM, homopolymer pAESO and copolymer series pAESO/VDM.

The chemical structure of the polymer pAESO and the polymer series pAESO/VDM was investigated by FTIR spectroscopy (Figure 5). The reduction of the acrylic group signal at 1637 cm^−1^ was observed in the FTIR spectra of photocross-linked polymers pAESO and all the polymers pAESO/VDM in comparison to that of the FTIR spectra of AESO and VDM showing that most of the acrylic groups were reacted. The more intensive signal of acrylic group C=C at 1630 cm^−1^ in FTIR spectra of pAESO/VDM could be assigned to the free VDM methacrylic groups. The strong peak at 1513 cm^−1^ is assigned to VDM aromatic ring C=C vibrations.

The formation of polymer cross-linked structure was confirmed by Soxhlet extraction. The yield of insoluble fraction of pAESO was much higher (88%) than that of the cross-linked polymers pAESO/VDM (31–63%) (Table 2). This showed that AESO tends to form densely cross-linked polymers even without photoinitiators. Sucha low yield of insoluble fraction of the polymer series pAESO/VDM could be explained by the tendency of VDM to form linear and/or branched polymers. This statement is confirmed by the increase of the yield of an insoluble fraction with the reduction of VDM amount.

### 3.3. Thermal Properties

DSC confirmed that all synthesized photocross-linked polymers are amorphous materials. Only the glass transitions were observed in the thermograms of all the polymers prepared. The values of the glass transition temperatures (T_g_) were very low and varied from −4.5 °C to −1.6 °C (Table 3) even though these polymers were solid materials at room temperature. Such low T_g_ values were determined by the flexible chains of AESO. The lower T_g_ value of the polymers pAESO/VDM1 and pAESO/VDM2 than pAVDM3 could be explained by their lower yield of insoluble fraction. A huge amount of linear and/or branched macromolecules was formed in these polymers probably due to the different activities of the functional group affected the T_g_ value of these copolymers [51]. The cross-linked structure of polymers caused their solid state at room temperature. Such a feature of the natural oil-based cross-linked polymers was observed in earlier studies [30,52].

**Figure 6:**
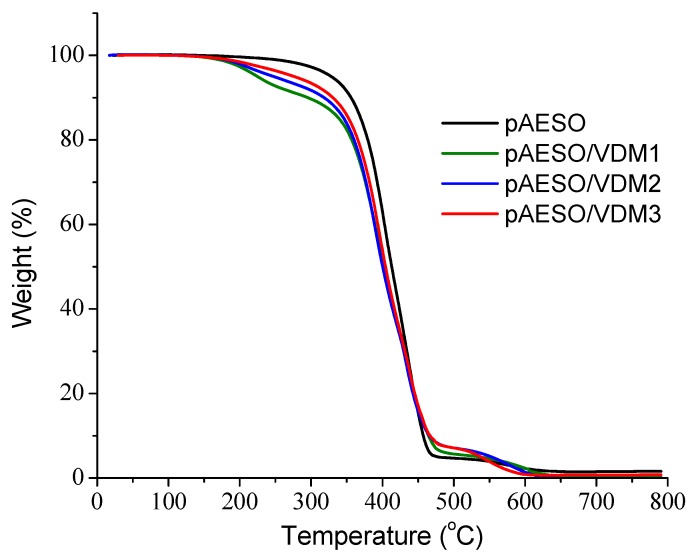
The thermogravimetric curves of the polymers pAESO and the polymer series pAESO/VDM.

TGA confirmed that the photocross-linked polymers exhibited high thermal stability. TGA curves of the cross-linked polymers are presented in Figure 6. The 10% weight loss temperatures (T_dec-10%_) of pAESO and the polymer series pAESO/VDM range from 295 °C to 356 °C (Table 3). It was noticed, that the higher the amount of VDM was used, the lower the T_dec-10%_ value indicated. Lower T_dec-10%_ values and a two- or even three-step thermal decomposition of the polymers containing VDM fragments could be due to the higher rate of linear and/or branched macromolecules while higher thermal stability is related to the high density of the cross-links [53].

### 3.4. Compressive Modulus

The compressive modulus of the synthesized cross-linked polymer samples was determined from the top pressure test. No visible cracks were observed after the testing of all polymer samples. The higher value of the compressive modulus was observed for the polymer pAESO in comparison to that of the polymers pAESO/VDM1 and pAESO/VDM2, and similar to that of the polymer pAESO/VDM3 (see Table 2). This shows the correlation of the compressive modulus and insoluble fraction of cross-linked polymers. The higher yield of the insoluble fraction caused the higher value of the compressive modulus. The tendency of the increase of the compressive modulus with the reduction of the VDM amount in the resin was also observed.

### 3.5. Characterization of DLW 3D Lithography Produced Structures

**Figure 7:**
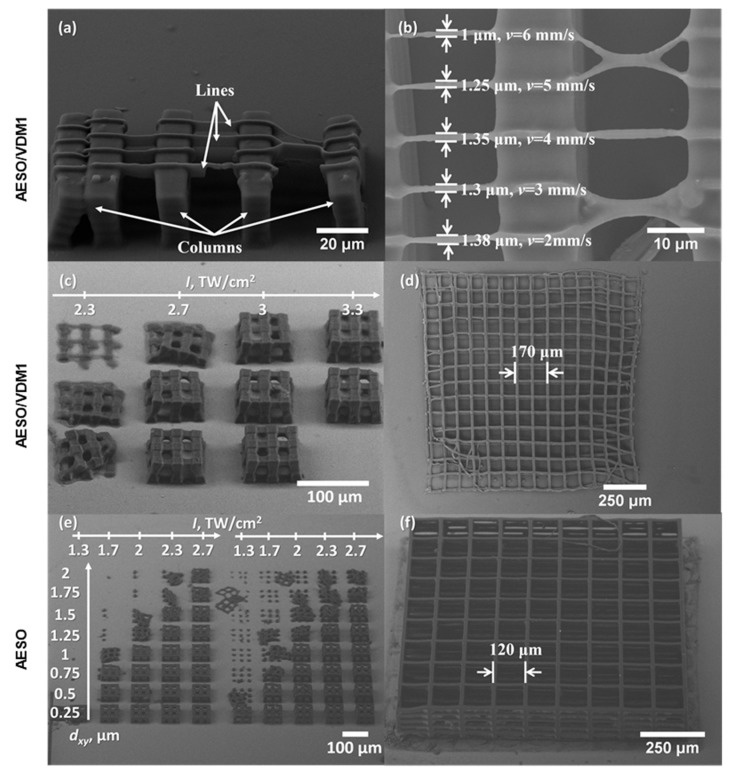
The SEM images of RB and 3D microporous woodpile structures: (**a**) a side view of RB at the angle of 45 degrees and 1800 magnification. The applied power *P* to produce bridges was 0.6 mW (2 TW/cm^2^), scan velocity *v* varied from 0.1 mm/s to 0.5 mm/s every 0.1 mm/s; (**b**) a top view of the other RB at 4000 magnification. *P* = 0.6 mW (2 TW/cm^2^), *v* = 2–6 mm/s every 1 mm/s; (**c**) 75 × 75 µm^2^ woodpile structures with a 30 µm period, *v* = 5 mm/s, the scale at the top of the image demonstrates the applied *I*; (**d**) a 1065 × 1065 µm^2^ woodpile with a 75 µm period, *v* = 5 mm/s, *P* = 0.4 mW (1.3 TW/cm^2^). (a–d) The structures were fabricated out of the resin AESO/VDM1; (**e**) 75 × 75 µm^2^ woodpile structures with a 30 µm period, *v* = 5 mm/s. Scale on the left of image shows the distance *d_xy_* between neighboring scans; (**f**) a 1095 × 1095 µm^2^ woodpile with 120 µm period, *v* = 5 mm/s, *P* = 0.6 mW (2 TW/cm^2^). (**e**,**f**) structures were fabricated out of AESO.

RB and 3D microporous woodpile structures obtained via DLW 3D lithography out of AESO and ASEO/VDM1 were characterized. These plant-derived resins were laser polymerized using ultrashort pulses by multiphoton absorption and avalanche induced cross-linking [54,55]. Scanning Electron Microscopy (SEM) images are shown in Figure 7. Figure 7a demonstrates RB—five columns with five straight lines perpendicular to the column long edges. Figure 7b shows a close up view of RB from the top. *P* was set to 0.6 mW (2 TW/cm^2^) for the lines and each line was scanned with a different velocity *v*, varying from 2 mm/s to 6 mm/s. It can be seen that each line has a slightly different width: the higher the *v*, the narrower the line. Further 3D microporous woodpile structures were formed. Arrays of 75 × 75 µm^2^ woodpiles are demonstrated in Figure 7c,e. In both cases, *v* was set to 5 mm/s and *P* was varied in the range of 0.4–1 mW (1.3–3.3 TW/cm^2^). It can be seen that an increased laser power *P* higher than *d_xy_* can be used. Finally, the mm scale woodpiles were fabricated. Figure 7d shows a 1065 × 1065 µm^2^ woodpile with a 75 µm period and 4 layers, *v* = 5 mm/s, *P* = 0.4 mW (1.3 TW/cm^2^). However, it did not sustain itself due to the low mechanical rigidity. Figure 7f demonstrates a 1095 × 1095 µm^2^ woodpile with a 120 µm period and 6 layers, *v* = 5 mm/s, *P* = 0.6 mW (2 TW/cm^2^). The structure sustained itself and had a 3D architecture. To sum up, AESO and the resin AESO/VDM1 can be the great candidates as new renewable materials for DLW 3D lithography technology [56]. It has a wide working window ranging *P* from 0.1 mW to 1 mW (dynamic fabrication window aspect ratio 10 times). Spatial features of 1 µm and a 6900 voxels/second throughput was achieved as a method for the evaluation of normalized µ-3D fabrication throughput [57]. We note that the possibility to the photostructure without the use of photoinitiators is not limited to serial direct write (DLW/SLA), but also can be extended to projection DLP lithography (SLM/DMD based) if the peak exposure intensity is sustained (for current case of employed material and light source the ranging within ≈ 1–3 TW/cm^2^).

It is envisaged that the photostructuring without the photoinitiators is beneficial for the fields of biomedicine, micro-optics and nanophotonics. The avoidance of toxic photoinitiators increases the integrity of biodegradable cell-growth scaffolds and reduces the auto-fluorescence while performing microscopy in vitro or in vivo. The absorbing materials are detrimental for the use in micro-optics and nanophotonics due to their reduced optical resilience and induced signal losses [58]. Moreover, the use of plant-derived materials in such technologies would benefit greatly due to their low toxicity, high biodegradability, and improved recycling options. Finally, it would reduce the dependency on limited and increasingly expensive fossil resources as well as greenhouse gas emission, which are the targets of the European Commission initiated “Europe 2020” strategy.

## 4. Conclusions

The real-time photorheometry study revealed the higher rate of photocross-linking of pure acrylated epoxidized soybean oil than that of its mixture with vanillin dimethacrylate or vanillin diacrylate without a photoinitiator and solvent. Novel plant-derived photocross-linked polymers were synthesized from acrylated epoxidized soybean oil and its mixtures with vanillin dimethacrylate. It was determined that the addition of vanillin dimethacrylate reduced the rate of photocross-linking and the values of the glass transition temperature, thermal decomposition temperature and compressive modulus. The formation of more linear and/or branched macromolecules considered the vanillin dimethacrylate effect as a plasticizer for acrylated epoxidized soybean oil in photocross-linking without a photoinitiator. It was experimentally demonstrated that the homopolymer of acrylated epoxidized soybean oil and the copolymer of acrylated epoxidized soybean oil and vanillin dimethacrylate are suitable materials for rapid 3D microstructuring by the direct laser writing lithography technique. Spatial features of 1 µm and a 6900 voxels/second throughput was achieved. Since the 3D cross-linking of the plant-derived materials was initiated using ultrafast laser inducing multiphoton absorption and avalanche ionization, it does not require the usage of any photoinitiator and opens a new pathway for green 3D µ-printing as a flexible tool for rapid prototyping or advanced additive manufacturing.

## Figures and Tables

**Table 1:** The composition of resins.

Resin	Ratio of Functional Groups ^1^	Ratio of Monomers, mol	Amount of Monomers, g
AESO	-	1	1.5
AESO/VDM1	1.5:1	1:1	1.5/0.375
AESO/VDM2	3:1	1:0.5	1.5/0.187
AESO/VDM3	6:1	1:0.25	1.5/0.094
AESO/VDA1	1.5:1	1:1	1.5/0.339
AESO/VDA2	3:1	1:0.5	1.5/0.170
AESO/VDA3	6:1	1:0.25	1.5/0.085

^1^ Ratio of AESO acrylic groups and VDM or VDA acrylic groups.

**Table 2:** The storage modulus (G’), loss modulus (G”), complex viscosity (η*), and gel time (t_gel_) of all resins.

Resin	G’ ^1^, Pa	G” ^1^, Pa	η* ^1^, mPa·s	t_gel_, s ^2^
AESO	4.76 × 10^6^	1.47 × 10^6^	7.93 × 10^4^	49
AESO/VDM1	2.65 × 10^6^	1.01 × 10^6^	4.51 × 10^4^	98
AESO/VDM2	2.70 × 10^6^	9.31 × 10^5^	4.55 × 10^4^	91
AESO/VDM3	3.51 × 10^6^	1.18 × 10^6^	5.89 × 10^4^	66
AESO/VDA1	5.89 × 10^5^	2.27 × 10^5^	1.01 × 10^4^	782
AESO/VDA2	1.11 × 10^6^	3.74 × 10^5^	1.87 × 10^4^	531
AESO/VDA3	3.56 × 10^6^	1.17 × 10^6^	5.96 × 10^4^	290

^1^ at an irradiation time of 600 s for AESO and AESO/VDM, and 1800 s for AESO/VDA. ^2^ calculated from the onset of UV/VIS irradiation.

**Table 3:** The yield of the insoluble fraction and the thermal and mechanical characteristics of the cross-linked polymers.

Polymer	Yield of Insoluble Fraction ^1^ (%)	T_g_^2^ (°C)	T_dec-10%_^3^ (°C)	E_c_^4^ (Pa)
**pAESO**	88	−4.5	356	0.62 ± 0.1
**pAESO/VDM1**	31	−2.6	295	0.19 ± 0.03
**pAESO/VDM2**	48	−2.6	318	0.46 ± 0.13
**pAESO/VDM3**	63	−1.6	331	0.66 ± 0.13

^1^ After Soxhlet extraction with chloroform for 24 h.; ^2^ Glass transition temperature estimated by DSC; ^3^ Temperature at the weight loss of 10 % obtained from TGA curves; ^4^ Compressive modulus from the top pressure test.
